# β-Lactam/β-Lactamase Inhibitor Combinations in Sepsis-Associated Acute Kidney Injury and Renal Replacement Therapy

**DOI:** 10.3390/antibiotics14111097

**Published:** 2025-11-01

**Authors:** Antonio Lacquaniti, Valentina Pistolesi, Antonella Smeriglio, Domenico Santoro, Cristina Iannetti, Giuseppe Lentini, Roberto Chimenz, Valeria Chirico, Domenico Trombetta, Santo Morabito, Paolo Monardo

**Affiliations:** 1 Nephrology and Dialysis Unit, Department of Internal Medicine, Papardo Hospital, 98158 Messina, Italy; 2 Hemodialysis Unit, Department of Internal Medicine and Clinical Sciences, Azienda Ospedaliero-Universitaria Policlinico Umberto I, Sapienza University of Rome, Viale del Policlinico, 155, 00161 Rome, Italy; valentina.pistolesi@uniroma1.it (V.P.); santo.morabito@uniroma1.it (S.M.); 3 Department of Chemical, Biological, Pharmaceutical and Environmental Sciences, University of Messina, 98122 Messina, Italy; 4 Unit of Nephrology and Dialysis, Department of Clinical and Experimental Medicine, University of Messina, 98125 Messina, Italy; 5 Pediatric Nephrology and Dialysis Unit, University Hospital “G. Martino”, 98124 Messina, Italy

**Keywords:** renal replacement therapy, sepsis-associated acute kidney injury, multidrug-resistant Gram-negative bacteria, β-lactam/β-lactamase inhibitor combinations, pharmacokinetics/pharmacodynamics, augmented renal clearance therapeutic drug monitoring, antibiotic dosing adjustment, critically ill patients, antimicrobial stewardship

## Abstract

Sepsis-associated acute kidney injury (SA-AKI) often requires renal replacement therapy (RRT), which markedly alters antimicrobial pharmacokinetics (PK) and pharmacodynamics (PD). Novel β-lactam/β-lactamase inhibitor (BL/BLI) combinations broaden options against multidrug-resistant Gram-negative bacteria, but dosing during RRT remains uncertain. This review summarizes PK/PD features, extracorporeal clearance, and practical dosing considerations about ceftolozane–tazobactam, ceftazidime–avibactam, aztreonam–avibactam, cefiderocol, meropenem–vaborbactam, imipenem–relebactam, and newer agents including sulbactam–durlobactam, cefepime–enmetazobactam, and cefepime–taniborbactam. Pharmacokinetic data, RRT impact, PK/PD targets, pediatric aspects, and clinical outcomes were extracted from experimental models, case reports, and clinical studies. Drug exposure varies with RRT modality, effluent flow, membrane properties, and patient-specific factors such as augmented renal clearance, hypoalbuminemia, and fluid overload. Standard renal-adjusted dosing often yields subtherapeutic concentrations in critically ill patients. Pediatric data remain scarce and largely limited to case reports. Optimal BL/BLI use in septic patients with SA-AKI on RRT requires individualized dosing that accounts for PK/PD variability and dialysis settings. Full-dose initiation during the first 24–48 h, followed by careful adjustment, appears prudent. Therapeutic drug monitoring should be used when available, and institution-specific protocols should be integrated into stewardship programs to improve efficacy and minimize resistance.

## 1. Introduction

Sepsis, a dysregulated host response to infection, is the leading cause of acute kidney injury (AKI) in critically ill patients. It represents a major healthcare concern, characterized by high incidence and mortality over the past four decades [1].

Worldwide, sepsis is estimated to affect more than 30 million individuals annually, potentially causing 6 million deaths [2]. In 2019, of an estimated 14 million infection-related deaths, 7 million were attributed to 33 bacterial pathogens (both antimicrobial-resistant and susceptible), accounting for 14% of all global deaths and 56% of all sepsis-related deaths. *Staphylococcus aureus*, *Escherichia coli*, *Streptococcus pneumoniae*, *Klebsiella pneumoniae*, and *Pseudomonas aeruginosa* were responsible for 55% of these events [3].

A cornerstone of severe sepsis management is the prompt initiation of appropriate antimicrobial therapy, the most critical determinant of reduced mortality and treatment costs [4]. However, administering antibiotics within a strict time frame for all patients with suspected sepsis requires substantial resources and may increase the risk of unnecessary broad-spectrum antibiotic exposure in non-infected patients, with potential adverse consequences [5]. Nevertheless, considerable controversy persists regarding the relationship between time to antibiotic administration and clinical outcomes in sepsis or septic shock, and whether treatment within one hour improves prognosis [6,7]. The Infectious Diseases Society of America suggests that aggressive antibiotic administration within one hour may not always be beneficial and can lead to inappropriate broad-spectrum use [8,9].

Antibiotic resistance is recognized as one of the greatest public health challenges of our time, threatening the continued availability of effective treatments for common infections. Multidrug-resistant (MDR) pathogens, including Gram-negative organisms such as carbapenemase-producing Enterobacteriaceae (CPE), MDR or extensively drug-resistant (XDR) *Pseudomonas aeruginosa*, and *Acinetobacter baumannii*, as well as Gram-positive organisms such as vancomycin-resistant *Enterococcus faecium* (VRE) and methicillin-resistant *Staphylococcus aureus* (MRSA), represent a healthcare concern.

Recent evidence indicates marked increases in the use of broad-spectrum agents such as carbapenems, third- and fourth-generation cephalosporins, and BL/BLI combinations. Coupled with data suggesting that up to 30% of prescriptions may be inappropriate, these trends imply that antibiotic resistance will remain a substantial future threat [10]. In this context, antibiotic dosing errors are frequent in severe sepsis and septic shock [11]. Pharmacokinetic studies in critically ill patients receiving various β-lactams have shown that plasma concentrations remained below the minimal inhibitory concentration (MIC) during 50% or even the entire dosing interval in 19% and 41% of patients, respectively [12].

This issue is largely explained by abrupt changes in glomerular filtration rate (GFR) and the frequent occurrence of AKI during sepsis. As a result, both increased and reduced renal clearance—leading to shortened or prolonged half-life—may occur during intensive care, rendering drug concentrations highly unpredictable and achievement of PK/PD targets difficult [13]. Furthermore, protein binding and volume of distribution (Vd) are affected by sepsis-associated conditions such as hypoalbuminemia and fluid overload.

In this scenario, the requirement for renal replacement therapy (RRT) adds another layer of complexity, making appropriate antibiotic dosing particularly challenging [14]. Evidence on the management of novel agents in AKI patients remains scarce, and available PK data during RRT are often limited to in vitro studies, rarely validated by in vivo analyses, complicating rational dosing.

This narrative review therefore focuses on recently introduced β-lactam/β-lactamase inhibitor (BL/BLI) combinations. We critically analyze the current literature to support clinicians in selecting optimal dosing regimens for critically ill patients with MDR infections, with special attention to those with sepsis-associated AKI (SA-AKI) requiring RRT.

In particular, a literature search was conducted on PubMed-MEDLINE evaluating the PK/PD data on BL/BLI in renal populations. “Ceftolozane–tazobactam, ceftazidime–avibactam, aztreonam–avibactam, cefiderocol, meropenem–vaborbactam, imipenem–relebactam, sulbactam–durlobactam, cefepime–enmetazobactam, and cefepime–taniborbactam, acute kidney injury, renal replacement therapy, continuous renal replacement therapy, continuous venovenous hemofiltration, continuous venovenous hemodialysis, continuous venovenous hemodiafiltration, pharmacokinetic, pharmacokinetic/pharmacodynamic, antibiotic infusion, therapeutic drug monitoring, pediatric antibiotic administration” were the terms searched on PubMed.

Pharmacokinetic data, RRT impact, PK/PD targets, pediatric aspects, and clinical outcomes were extracted from experimental models, case reports, and clinical studies.

## 2. Pharmacokinetics Changes in Sepsis and AKI

The altered immune response of critically ill patients and the differences in pharmacokinetics (PK) and pharmacodynamics (PD) of antimicrobials contribute to the challenge of optimizing antimicrobial therapy.

When acute kidney injury (AKI), with or without the need for renal replacement therapy (RRT), complicates sepsis, dosing management becomes even more complex due to rapid fluctuations in renal and extracorporeal clearance. This increases the risk of either drug accumulation with toxicity or underdosing with consequent treatment failure and resistance development [15]. Dynamic changes in residual renal function and the specific clinical context (e.g., sepsis, polytrauma) help explain why the PK behavior of some antimicrobials in critically ill patients differs markedly from that of healthy controls matched for creatinine clearance and body weight [16,17].

In the early stages of sepsis, augmented renal clearance (ARC) may occur, substantially increasing the risk of antibiotic underexposure. ARC is increasingly recognized in specific subgroups of critically ill patients, with an incidence ranging from 14% to 80% [18]. Patients with ARC show significantly increased clearance of β-lactams, and several studies have reported that standard dosing often yields trough concentrations below the pathogen’s MIC. Consequently, dosing adjustments for renally cleared antimicrobials must account for ARC to avoid subtherapeutic exposure [19,20]. Estimating renal function by serum creatinine is unreliable in the ICU because of fluid shifts and altered muscle mass; therefore, direct measurement of creatinine clearance using timed urine collections (2–24 h) should be encouraged [21].

The volume of distribution (Vd) of many drugs, including antimicrobials, is frequently increased in critically ill patients due to fluid resuscitation, drug infusions, blood products, and parenteral nutrition. Hydrophilic antibiotics such as β-lactams require higher loading doses to offset this expanded Vd and achieve therapeutic levels. Protein-binding alterations further complicate drug distribution: hypoalbuminemia increases the free drug fraction, facilitating redistribution into the interstitial space and modifying clearance through the liver, kidneys, and RRT. Because unbound drug levels are more variable in septic patients with hypoalbuminemia, interpretation of therapeutic drug monitoring (TDM) should be cautious when only total concentrations are available [22,23,24].

Severe AKI requiring RRT adds another layer of complexity, as the relative contribution of residual renal clearance and extracorporeal clearance to total body clearance may fluctuate over time [25].

Drug-related factors, such as molecular weight (MW), Vd, and protein binding, as well as dialysis-related factors such as filter type, blood flow rate, effluent rate, and reinfusion modality, all influence extracorporeal clearance. Most antimicrobials have MW < 1000 Da and are readily removed by diffusive techniques, as diffusion inversely correlates with MW. In convective modalities, the sieving coefficient (SC)—the ratio of solute concentration in ultrafiltrate to plasma water—approximates the free fraction for drugs with MW up to 1500 Da. Drugs with Vd < 1 L/kg are significantly affected by extracorporeal clearance, while those with Vd > 2 L/kg are minimally influenced because of extensive tissue distribution. Only the free (unbound) fraction of a drug is subject to extracorporeal removal, and SC correlates closely with this fraction. Thus, antimicrobials with high MW, high protein binding (>80%), and large Vd (>2 L/kg) are least affected by RRT [26].

By contrast, β-lactams, characterized by hydrophilicity, low Vd, and predominant renal clearance, are highly susceptible to extracorporeal removal. In critically ill patients undergoing both diffusive and convective RRT modalities, β-lactam exposure can vary widely if dialysis prescriptions are not standardized [27]. Moreover, membrane characteristics (pore size, adsorptive capacity), blood and effluent flow rates, and RRT modality (intermittent hemodialysis [IHD], prolonged intermittent RRT [PIRRT], or CRRT) strongly impact drug clearance. While CRRT provides relatively constant clearance under stable conditions, IHD and PIRRT create alternating phases, with rapid intradialytic removal followed by slower interdialytic kinetics. High-flux membranes (cutoff ~20,000 Da) remove even larger molecules (>1000 Da) more effectively than low-flux membranes, and different membrane compositions (e.g., polysulfone, polyacrylonitrile, polymethylmethacrylate) vary in adsorptive properties. The integration of selective or non-selective adsorptive devices further complicates dosing, and studies are warranted to evaluate antibiotic adsorption in this context [28].

Since β-lactams exert time-dependent bactericidal activity, their efficacy depends on the fraction of the dosing interval during which free drug concentrations exceed the MIC (%fT > MIC). While bactericidal activity generally requires 40–70% fT > MIC, emerging data suggest that more aggressive targets (approaching 100% fT > 4–5 × MIC) may be advantageous in critically ill patients [22,29]. For cephalosporins, maintaining unbound concentrations above the MIC for at least 40% of the interval is standard, but 100% is desirable in critically ill populations. For *Pseudomonas aeruginosa*, EUCAST and CLSI define an MIC breakpoint of 4 mg/L [30]. For newer BL/BLI agents, the PK/PD index most closely associated with efficacy is fT > MIC, and consensus recommendations suggest aiming for 100% fT > MIC, though this must be balanced against toxicity risks and tailored to individual patients [22,31,32,33].

Currently, dosing recommendations for novel BL/BLIs exist for patients receiving IHD but remain undefined for those undergoing continuous RRT, underscoring the urgent need for clinical data in this setting.

## 3. Antibiotics

Hydrophilicity, low plasma protein binding, small volume of distribution (Vd), low molecular weight (MW), and predominant renal clearance characterized all β-lactam/β-lactamase inhibitor (BL/BLI) antibiotics, necessitating daily dose adjustments in the presence of kidney dysfunction or during an RRT [27].

The optimal PK/PD index varies across BLs and BLIs. For β-lactams, efficacy correlates with the fraction of the dosing interval during which free drug concentrations exceed the MIC (%fT > MIC). For most BLIs, activity is instead linked to the percentage of the interval in which concentrations exceed a defined threshold (CT), which varies depending on the inhibitor. For relebactam and vaborbactam, efficacy is best described by the ratio of the unbound area under the concentration–time curve to the MIC (fAUC/MIC) [34]. Importantly, both the BL and its BLI must achieve their respective PK/PD targets simultaneously for the combination to be effective.

Recent evidence suggests that more aggressive PK/PD targets for β-lactams (up to 100% fT > 4–5 × MIC) may be necessary to minimize microbiological failure and resistance, particularly in critically ill patients [35].

When prescribing antimicrobials during RRT, four main factors must be considered: (i) drug-related PK/physicochemical properties, (ii) RRT modality, settings, and filter type, (iii) infection site and pathogen MIC, and (iv) critical illness-associated PK alterations.

### 3.1. Ceftolozane–Tazobactam

Ceftolozane–tazobactam (C/T) is a novel cephalosporin/β-lactamase inhibitor combination unaffected by the most common resistance mechanisms, including efflux pumps, reduced porin uptake, and altered penicillin-binding proteins. It is considered a first-line option for *Pseudomonas aeruginosa* infections [36]. Both components are eliminated by the kidneys; in patients with reduced glomerular filtration rate (GFR), drug accumulation occurs due to prolonged half-life, requiring dose reduction [37]. C/T exhibits linear PK in patients with preserved renal function but prolonged half-lives in renal impairment [38]. Ceftolozane has a molecular weight of 666 Da and ~20% protein binding [33], properties that predict significant clearance during CRRT.

C/T shows low protein binding and hydrophilic properties, making it readily removable by RRT [39]. A strong positive correlation has been demonstrated between RRT intensity (effluent flow rate) and ceftolozane clearance, regardless of modality, while blood flow rate (Qb) has a minimal impact [40]. These findings reflect ceftolozane’s physicochemical features: low MW, low protein binding, small Vd, and hydrophilicity.

Preliminary reports suggested that standard dosing (1.5 g q8h) ensures adequate C/T exposure in patients on RRT [41,42]. However, this regimen—sufficient for effluent flow rates of 1200–2700 mL/h—may be inadequate to reach PK/PD targets, particularly in patients with residual diuresis. Therefore, full-dose administration (3 g q8h) should be considered in patients with deep-seated infections (e.g., pneumonia) or when *P. aeruginosa* isolates exhibit elevated ceftolozane MICs (>4 mg/L) [43].

PK data in children and adolescents are limited, and no studies have investigated C/T in pediatric patients undergoing CRRT. A single case report described successful treatment of sepsis caused by MDR *P. aeruginosa* with a regimen of 35 mg/kg q8h [44].

### 3.2. Ceftazidime–Avibactam

Ceftazidime–avibactam (CZA) combines a third-generation cephalosporin with avibactam, a non-β-lactam β-lactamase inhibitor that targets Ambler class A, C, and some class D enzymes (e.g., KPC and OXA-48). Avibactam is inactive against class B metallo-β-lactamases (MBLs, e.g., VIM, IMP, NDM) and certain OXAs in *Acinetobacter* spp. The recommended dose in adults with normal renal function is 2 g/0.5 g q8h as a 2 h infusion [45,46].

FDA-approved dosing for patients with CLCr < 15 mL/min is 0.94 g q24–48h [47], but no specific guidance exists for patients on RRT. PK data are limited to case reports. Wenzler et al. demonstrated that 1.25 g q8h maintained free ceftazidime and avibactam levels above the MIC throughout the dosing interval during CVVH [48]. Conversely, Soukup et al. reported that only full dosing (2.5 g q8h) achieved high trough concentrations (>32 mg/L) during CVVHDF [49]. Thus, 1.25 g q8h may suffice for susceptible strains (MIC < 4 mg/L), while higher regimens are warranted for less susceptible isolates. In patients with CRE infections treated with CZA, RRT was an independent predictor of resistance development [50,51].

CZA was FDA-approved for children ≥3 months in 2019. Clinical experience is limited to case series: in one cohort of 38 children with CRO infections, CZA achieved an 84% success rate, though only 3 required RRT and no dosing data were provided [52,53,54,55]. In another series of 37 pediatric patients, two underwent hemodialysis, again without details on dosing or PK/PD management [56]. Importantly, avibactam is ineffective against MBL producers, and resistance rates vary across studies (36–75%) [57,58].

### 3.3. Aztreonam–Avibactam

Aztreonam–avibactam (AZA) combines aztreonam, stable against MBL hydrolysis, with avibactam, which protects it from co-produced serine β-lactamases. This makes AZA particularly valuable against MBL-producing Enterobacterales. It is under development for complicated intra-abdominal infections, hospital-acquired pneumonia, and ventilator-associated pneumonia [59,60,61,62,63,64,65].

PK/PD targets are derived from preclinical models: maintaining free aztreonam above 8 mg/L for 60% of the interval and free avibactam above 2.5 mg/L for 50% [66,67]. Phase 3 regimens included loading followed by extended 3 h infusions q6h, with renal adjustments. However, no patients on RRT were included in pivotal trials [68]. Thus, no validated dosing exists for CRRT or IHD populations.

AZA shows promise for pediatric CRKP infections, especially in regions with a high prevalence of NDM carbapenemase genes [59,69,70]. Since avibactam cannot inhibit MBLs alone, AZA is the only BL/BLI active against these pathogens. Pediatric PK modeling suggests activity, but no RRT-specific data are available.

### 3.4. Cefiderocol

Cefiderocol (CEFI) is a siderophore cephalosporin that uses iron transport systems to cross bacterial membranes, showing potent activity against carbapenem-resistant Enterobacterales and non-fermenters, including *Acinetobacter baumannii* and *Stenotrophomonas maltophilia*. Protein binding is 40–60% [71]. Its PK/PD driver is %fT > MIC, with target ranges varying across pathogens (53–88%) [72]. CEFI is primarily excreted renally with minimal metabolism, and half-life is prolonged in renal impairment [73].

CEFI is the first novel BL/BLI with manufacturer dosing recommendations for CRRT: 1.5 g q12h to 2 g q8h [74]. PK predictions were extrapolated from cefepime, adjusted for protein binding. Extended (3 h) infusions are recommended. Doses of 2 g q8h yield high plasma exposures even in septic CRRT patients, though adverse events are generally mild. The probability of target attainment exceeds 90% against pathogens with MIC ≤ 4 mg/L with these regimens [75,76,77].

Although the drug is approved only for adults, pediatric PK modeling suggests that 60 mg/kg q8h (3 h infusion) achieves adult-equivalent exposures in patients aged 3 months–18 years [78]. The PEDI-CEFI trial is ongoing [79]. A recent case report in a 16-year-old on CVVHDF plus CytoSorb^®^ hemoadsorption showed significant CEFI removal, requiring dosing adjustments and TDM [80].

### 3.5. Meropenem–Vaborbactam

Meropenem–vaborbactam (MVB) combines meropenem with vaborbactam, a boronic acid β-lactamase inhibitor active against KPC-producing Enterobacterales [81,82]. Vaborbactam exhibits ~33% protein binding, wide tissue distribution, and predominant renal excretion. Neither agent undergoes significant metabolism [83,84].

Data are limited to case reports and ex vivo models [85,86]. A recent population PK study of CRRT patients with CRE infections found a 54% reduction in clearance compared to patients with normal renal function, leading to vaborbactam accumulation [87,88]. PTA analysis suggested that 4 g q8h provides adequate coverage for MIC ≤ 1 mg/L, while 2 g q8h may suffice in CRRT patients. However, achieving fAUC/MIC > 38 for vaborbactam against high-MIC organisms remains difficult.

Although the drug is approved only for adults, pediatric experience is limited to case reports of intra-abdominal CRE infections in adolescents (11–15 years), showing safety and efficacy [89]. PK extrapolations suggest that 40 mg/kg q8h (≥3 months) yields ≥ 90% PTA, comparable to adult dosing.

### 3.6. Imipenem–Relebactam

Imipenem–relebactam (IMI/REL) combines imipenem/cilastatin with relebactam, a diazabicyclooctane β-lactamase inhibitor. Both exhibit dose-proportional PK, ~20% protein binding, and renal excretion [90,91]. Phase 2 trials demonstrated non-inferiority to standard regimens in complicated intra-abdominal and urinary tract infections, with better safety compared to colistin-based regimens [92].

Reduced dosing is recommended for hemodialysis patients (IMI 200 mg + REL 100 mg). No clinical data exist for critically ill patients on CRRT [93,94,95]. Ex vivo bovine models showed significant removal of both drugs by RRT, with clearance depending on effluent flow rates [96]. Simulations suggested that IMI 200 mg q6h achieves fT > MIC targets in 90% of virtual patients, but higher doses (600 mg q6h) are needed for stricter PK/PD goals (fT > 4 × MIC). REL consistently achieved target attainment across tested regimens.

IMI/REL has shown >90% in vitro activity against *E. coli*, *P. aeruginosa*, *K. pneumoniae*, and *E. cloacae* isolates from pediatric patients in the SMART surveillance program [97,98]. Clinical pediatric dosing regimens are not yet established.

Table 1 summarizes the key pharmacokinetic and pharmacodynamic characteristics, RRT-related clearance profiles, and dosing considerations of the most recently introduced β-lactam/β-lactamase inhibitor combinations, providing a practical overview to support antibiotic optimization in critically ill patients.

## 4. BL/BLIC Incoming

The above-mentioned beta-lactam/beta-lactamase inhibitor combinations licensed are not expected to be a definitive solution for managing the difficult-to-treat resistant (DTR) Gram-negative pathogens.

Metallo-beta-lactamase-producing Enterobacterales or *Pseudomonas aeruginosa* or carbapenem-resistant *Acinetobacter baumannii* could not respond to these therapies, and the rate of resistance against these agents is developing and increasing.

As revealed for the previously mentioned BL/BLI, the renal elimination represents the main route of catabolism of these agents. In patients with renal dysfunction, dosing adjustments are needed.

In septic patients with AKI, aggressive PK/PD target attainment is required, hypothesizing that a rapid recovery of kidney function occurs in the first 48–72 h of renal impairment [99].

### 4.1. Sulbactam–Durlobactam

#### 4.1.1. Structure, Chemical Characteristics, and Clinical Indication

Durlobactam (DUR) is a β-lactamase inhibitor of class A, C, and D enzymes and restores sulbactam susceptibility to *A. baumannii* strains. If administered alone, no activity has been observed, whereas when combined with sulbactam, it acts against *A. baumannii* [100,101].

The hospital-acquired and ventilator-associated bacterial pneumonia caused by susceptible isolates of the *Acinetobacter baumannii*–calcoaceticus complex represents the main indication for the administration of sulbactam–durlobactam. In particular, the referred dose is 1 g of SUL and 1 g of DUR (1 g-1 g) q6h administered as a 3 h infusion in patients with GFR > 50 mL/min. This condition is secondary to a renal elimination of the drug. For this reason, dosage modifications are recommended for patients with moderate-to-severe renal dysfunction, including those requiring intermittent hemodialysis [102].

#### 4.1.2. Prescription and RRT

SUL and DUR adsorption, protein binding, and transmembrane clearance for different hemofilters, such as M100 and HF1400, were assessed in an ex vivo RRT model using bovine blood.

Two dosing regimens were suggested according to the effluent flow rate, whereas hemofilter type, RRT mode, such as CVVH or CVVHD, or point of replacement fluid dilution did not alter the PK and PD properties of SUL-DUR. In particular, a dose of SUL-DUR 1 g-1 g q8h with each dose administered as a 3 h infusion was recommended for effluent flow rates < 3 L/h, whereas the same dosage but every six hours was suggested if the effluent flow rates were 3–5 L/h [103].

Only one report describes the in vivo PK evaluation of SUL-DUR in a patient with carbapenem-resistant Acinetobacter baumannii bacteremia and ventilator-associated bacterial pneumonia treated by CVVH with an effluent rate of 6 L/h. In particular, a dosage of 1 g-1 g every 4 h (3 h infusion) achieved PD targets for this isolate with an MIC of 4/4 mcg/mL, obtaining PK/PD targets with 100% free time above MIC (fT > MIC) [104].

### 4.2. Cefepime–Enmetazobactam

Enmetazobactam, a novel penicillanic acid sulphone BLI similar in structure to tazobactam but with higher potency and administered in combination with cefepime, mostly targets ESBLs [105].

*Escherichia coli*, *Klebsiella pneumoniae*, *Pseudomonas aeruginosa*, *Proteus mirabilis*, and *Enterobacter cloacae* infections represent the main indication of this drug [106], indicating that enmetazobactam does not have a direct antimicrobial effect [107].

In 2004, the Food and Drug Administration (FDA) approved the indication of cefepime/enmetazobactam for adults with complicated urinary tract infections (cUTIs) including pyelonephritis. Conversely, hospital-acquired pneumonia (HAP) and ventilator-associated pneumonia (VAP) were the indication approved in Europe [102].

Enmetazobactam has urinary excretion, and only <5% is bound to plasma proteins. The recommended frequency of administration of cefepime/enmetazobactam is every 8 h, but if eGFR is >60, the dosage should be adjusted by administering 1 g/0.25 g of the antibiotic every 8 h. When the eGFR is 15–29 mL/min/1.73 m^2^, the antibiotic should be administered every 12 h in a reduced dose of 1 g/0.25 g. Moreover, when the eGFR is <15 mL/min/1.73 m^2^ or in HD patients, cefepime/enmetazobactam is administered once per day in the same dose of 1 g/0.25 g. For CRRT patients, the recommended therapeutic dose is higher than the dose in HD patients.

### 4.3. Cefepime–Taniborbactam

Cefepime–taniborbactam could be a valuable option against infections caused by metallo-beta-lactamase-producing *Enterobacterales* or *Pseudomonas aeruginosa*.

The behavior of these drugs during CRTT has not been evaluated in clinical trials.

Recently, an ex vivo study using bovine whole blood evaluated cefepime in combination with taniborbactam across different CRRT modalities and various effluent flow rates, as well as at different points of fluid replacement. In particular, while the hemofilter type and CRRT mode were also significant predictors of clearance modifications, the effluent flow rate was the main influencer. A dose of 1 g–0.25 g q8h administered as a 4 h infusion for effluent flow rates < 3.5 L/h provided a similar AUC24h to a standard dosage. The full pneumonia dose of 2 g–0.5 g q8h as a 4 h infusion regimen provided similar AUC24h and optimal PTA for effluent flow rates of 3.5 to 5 L/h. Additionally, 2 g–0.5 g q12h (4 h infusion) retained excellent PTA and similar AUC24h at 3 and 4 L/h effluent rates and could be an option in that range [108].

## 5. BL/BLI Dosing Algorithm in Sepsis-Associated AKI Under RRT

Based on the available evidence, a stepwise algorithm has been developed to optimize the dosing of novel BL/BLI combinations in patients with sepsis-associated AKI undergoing RRT. The algorithm integrates PK/PD principles, RRT-related parameters, and patient-specific variables to ensure adequate therapeutic exposure while minimizing the risk of treatment failure and resistance.

1. Initial Phase (0–48 h): It is advisable to avoid an empirical dose reduction, whereas it appears reasonable to initiate a full standard dose, including a loading dose and extended/continuous infusion when feasible. This strategy is justified by the high variability of renal and extracorporeal clearance in the early stage, where underexposure increases the risk of treatment failure.
2. RRT Characterization: The RRT modality (IHD, PIRRT, CRRT: CVVH, CVVHD, CVVHDF) should be identified. Effluent flow rate, the main determinant of extracorporeal clearance, should be recorded, along with membrane type and potential use of adsorptive devices.
3. Drug- and Patient-Specific Factors: BL/BLI physicochemical properties (hydrophilicity, low protein binding, small Vd, resulting in high extracorporeal clearance) should be considered. Residual renal clearance or ARC should be assessed, and the infection site as well as the pathogen MIC should be evaluated.
4. Practical Dosing Considerations:
  - C/T: Standard 1.5 g q8h is often insufficient in CRRT; 3 g q8h may be required for high MIC or deep-seated infections.
  - CZA: 1.25–2.5 g q8h depending on MIC and effluent; dose fractionation is preferred over interval extension.
  - AZA: Limited RRT data; high clearance expected; extended infusion and higher dosing recommended.
  - FDC: Manufacturer guidance for CRRT ranges from 1.5 g q12h to 2 g q8h, with 3 h infusion; TDM is advised.
  - MVB: Risk of vaborbactam accumulation in CRRT; regimens vary from 2 to 4 g q8h depending on MIC and effluent.
  - IMR: Ex vivo data indicate strong extracorporeal removal; simulated dosing suggests 200–600 mg IMI q6h + 100 mg REL q6h depending on PK/PD targets.
5. Adjustment for RRT Modality:
  - CRRT: Full dosing should be maintained, with increased dose or frequency at higher effluent rates (≥35 mL/kg/h).
  - PIRRT: A loading dose should be administered before the session, with possible supplemental dosing afterward.
  - IHD: Supplemental dosing should be administered post-dialysis for hydrophilic BL with low protein binding and low Vd.
6. TDM: TDM should be performed whenever available. The target is 100% fT > MIC; in severe infections, 100% fT > 4–5 × MIC may be considered. Adjustments should primarily involve increasing dose or frequency rather than prolonging the dosing interval.
7. Daily Reassessment: RRT settings, residual renal function, pathogen MIC, clinical response, and potential toxicity should be re-evaluated daily. De-escalation should only be undertaken when both renal function and RRT prescription are stable.

**Figure 1:**
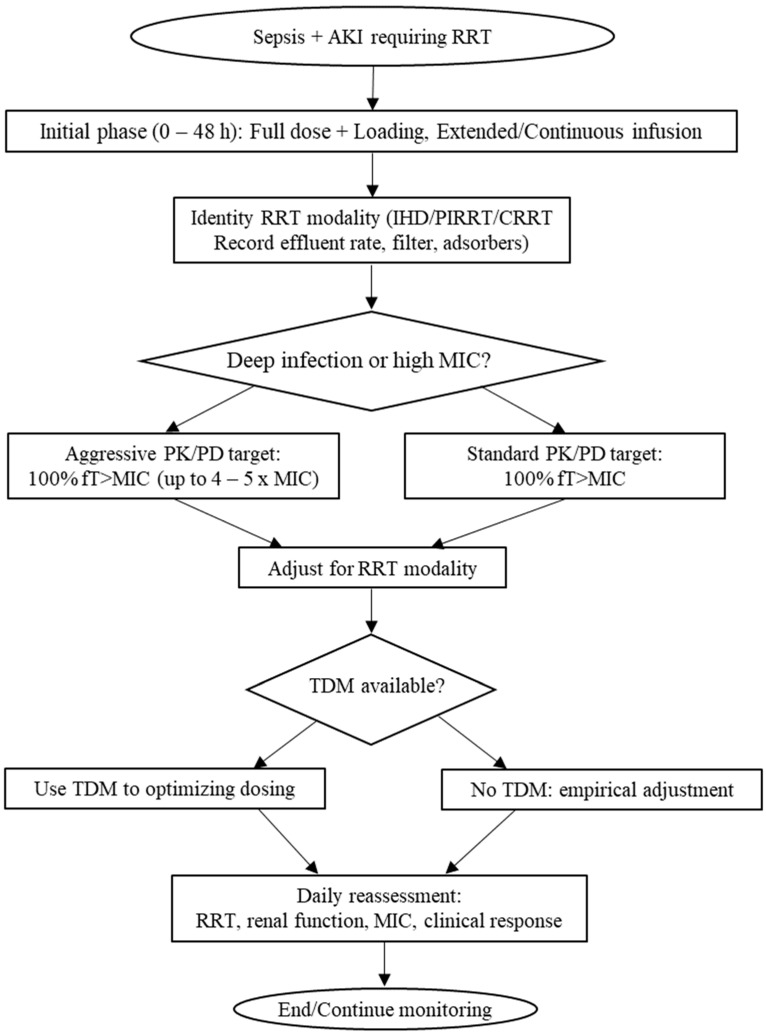
Flowchart summarizing BL/BLI dosing considerations in sepsis-associated AKI on RRT. All data are suggested but not recommended by clinical trials. Abbreviations: AKI: acute kidney injury; RRT: renal replacement therapy; IHD: intermittent hemodialysis; PIRRT: prolonged intermittent renal replacement therapy; CRRT: continuous renal replacement therapy; MIC: minimum inhibitory concentration; PK/PD: pharmacokinetics/pharmacodynamics; fT: free time; TDM: therapeutic drug monitoring.

A flowchart (Figure 1) has been designed to summarize this algorithm, integrating RRT modality, effluent rate, drug properties, and PK/PD targets, thereby providing a pragmatic visual guide for clinical decision-making in the dosing of novel BL/BLI combinations in septic patients with AKI receiving RRT.

## 6. Discussion

Management of severe infections in septic patients with acute kidney injury requiring renal replacement therapy represents a major clinical challenge, particularly when using novel β-lactam/β-lactamase inhibitor combinations. Based on the current evidence, several practical points can be highlighted:

(i) Avoid empirical dose reductions in the early phase of sepsis-associated AKI. During the first 24–48 h, recovery of renal function is unpredictable and subtherapeutic dosing may compromise outcomes; therefore, initial full-dose regimens should be maintained.

(ii) Consider RRT settings as key determinants of antibiotic clearance. Effluent flow rate, modality (CVVH, CVVHD, CVVHDF, PIRRT, IHD), and filter type strongly influence extracorporeal drug removal and must be integrated into dosing decisions; in particular, whereas scarce data are available about RRT modality and hemofilters, no suggestions could be proposed about adsorbers, which are widely applied in septic patients. The only results concern the cytosorb adsorber and cefiderocol, with negative effects in terms of PK/PD target achievement. Clinical data and TDM analyses are required.

(iii) Target aggressive PK/PD goals. In critically ill patients, aiming for 100% fT > 4–5 × MIC for β-lactams is advisable to reduce microbiological failure and prevent resistance; it has been demonstrated that RRT is an independent risk factor for drug resistance, suggesting that it may lead to inadequate drug exposure with non-negligible consequences in terms of stewardship implications. While renal dysfunction increases the risk of β-lactam overdosing, therapeutic failures have been observed, indicating the failure of PK/PD target achievement. Moreover, the recommendations for dose adjustment in cases of renal dysfunction are based on data from patients with chronic kidney disease, without comparison to critically ill patients. Furthermore, no data are available in terms of side effects occurring during RRT, which may result from the high risk of undertreatment due to drug removal rather than from drug accumulation. Moreover, the impact of these drugs on kidney function and the related risk of AKI is very low, and patients with serious infections need to receive effective therapies in a timely fashion, maximizing clinical response with minimal adverse events. In particular, the only datum derived from randomized trials pertains to the CEFI, the only new β-lactam for which high-dose schemes were used in patients with ARC. However, further data are needed for other BL/BLI combinations in this population.

(iv) Use TDM whenever available. Direct measurement of drug concentrations remains the most reliable approach to optimize therapy; when unavailable, dosing should be guided by infection severity, pathogen MIC, and local epidemiology.

(v) Integrate dosing strategies into antimicrobial stewardship programs. Institution-specific guidelines should incorporate RRT modalities, pharmacokinetic principles, and local resistance patterns to support clinicians in real-time decision-making. However, many dosing recommendations are derived from case reports, ex vivo models, or population PK simulations, and this limitation should be highlighted. Moreover, in the pediatric field, no robust data are available, and the scarcity of the results is related to case reports and observations derived from other antibiotics with similar characteristics to BL/BLIs. For these reasons, clinical trials enrolling pediatric patients requiring RRT are necessary to obtain strong dosing recommendations.

In conclusion, antibiotic dosing in septic patients on RRT requires a proactive and individualized approach. Until robust outcome data becomes available, clinicians should combine full-dose initiation, RRT-specific adjustments, and, where possible, TDM, to maximize efficacy while minimizing resistance and toxicity.

## Figures and Tables

**Table 1:** Key pharmacokinetic/pharmacodynamic parameters and dosing considerations of novel β-lactam/β-lactamase inhibitor combinations during renal replacement therapy (RRT).

Antibiotic Combination	MW (Da)	Protein Binding (%)	Vd (L/kg)	Renal Clearance	RRT Dosing Considerations
Ceftolozane–Tazobactam	666/300	~20	0.2–0.3	Predominant	Standard 1.5 g q8h often insufficient; consider 3 g q8h in CRRT, deep infections
Ceftazidime–Avibactam	546/265	~10	0.2–0.3	Predominant	1.25 g q8h may suffice (MIC < 4 mg/L); higher doses in CRRT or high MIC
Aztreonam–Avibactam	435/265	<10	0.2–0.3	Predominant	No RRT data; extrapolate from aztreonam + CZA; high clearance expected
Cefiderocol	751	40–60	0.2–0.3	Predominant	Manufacturer: 1.5 g q12h–2 g q8h; TDM advised in CRRT
Meropenem–Vaborbactam	383/297	2/33	0.3	Predominant	4 g q8h adequate for MIC ≤ 1 mg/L; accumulation risk of vaborbactam in CRRT
Imipenem–Relebactam	299/310	~20	0.2–0.3	Predominant	Ex vivo/model-based data: 200–600 mg IMI q6h; 100 mg REL q6h; clearance depends on effluent
Sulbactam–Durlobactam	233/348	<40	0.2–0.3	Predominant	q8h for effluent <3 L/h; q6h if 3–5 L/h; case: q4h at 6 L/h
Cefepime–Enmetazobactam	480/300	<10	0.2–0.3	Predominant	1 g/0.25 g q12–24h if eGFR < 30; higher doses in CRRT
Cefepime–Taniborbactam	480/314	<20	0.2–0.3	Predominant	Ex vivo/model-based data: dose tailored to effluent flow; q8h or q12h regimens

Abbreviations: MW, molecular weight; Vd, volume of distribution; RRT, renal replacement therapy; CRRT, continuous renal replacement therapy; MIC, minimum inhibitory concentration; CZA: Ceftazidime–Avibactam; TDM, therapeutic drug monitoring; eGFR, estimated glomerular filtration rate; IMI, imipenem; REL, relebactam. Notes: TDM for all antibiotics is recommended when RRT is prescribed. No data are available for PIRRT and IHD. However, a loading dose should be administered before a session of prolonged Intermittent Renal Replacement Therapy (PIRRT), with a possible supplemental dosing afterward, whereas a supplemental dosing should be administered post intermittent hemodialysis (IHD).

## Data Availability

Data sharing is not applicable.

## Abbreviations

The following abbreviations are used in this manuscript:

AKI	Acute Kidney Injury
ARC	Augmented Renal Clearance
AZA	Aztreonam–Avibactam
BL	Beta-Lactam
BL/BLI	Beta-Lactam/Beta-Lactamase Inhibitor
BLI	Beta-Lactamase Inhibitor
CEFI	Cefiderocol
CLSI	Clinical and Laboratory Standards Institute
CPE	Carbapenemase-Producing Enterobacteriaceae
CRE	Carbapenem-Resistant Enterobacterales
CRKP	Carbapenem-Resistant Klebsiella pneumoniae
CRO	Carbapenem-Resistant Organism
CRRT	Continuous Renal Replacement Therapy
CT	Concentration Threshold
CVVH	Continuous Veno-Venous Hemofiltration
CVVHDF	Continuous Veno-Venous Hemodiafiltration
CZA	Ceftazidime–Avibactam
DOI	Digital Object Identifier
EUCAST	European Committee on Antimicrobial Susceptibility Testing
FDA	Food and Drug Administration
fT	Free Time
GFR	Glomerular Filtration Rate
ICU	Intensive Care Unit
IHD	Intermittent Hemodialysis
IMI	Imipenem
IMI/REL	Imipenem–Relebactam
IMP	Imipenemase (Metallo-Beta-Lactamase class)
KPC	Klebsiella pneumoniae Carbapenemase
MBL	Metallo-Beta-Lactamase
MDR	Multidrug-Resistant
MIC	Minimum Inhibitory Concentration
MRSA	Methicillin-Resistant Staphylococcus aureus
MVB	Meropenem–Vaborbactam
MW	Molecular Weight
NDM	New Delhi Metallo-Beta-Lactamase
OXA	Oxacillinase (Class D Beta-Lactamase)
PD	Pharmacodynamics
PEDI	Pediatric Cefiderocol Trial (PEDI-CEFI)
PIRRT	Prolonged Intermittent Renal Replacement Therapy
PK	Pharmacokinetics
PK/PD	Pharmacokinetics/Pharmacodynamics
PTA	Probability of Target Attainment
REL	Relebactam
RRT	Renal Replacement Therapy
SA	Sepsis-Associated
SC	Sieving Coefficient
SMART	Study for Monitoring Antimicrobial Resistance Trends
TBCL	Total Body Clearance
TDM	Therapeutic Drug Monitoring
TRCL	Total Renal Clearance

## References

[1] Singer M., Deutschman C.S., Seymour C.W., Shankar-Hari M., Annane D., Bauer M., Bellomo R., Bernard G.R., Chiche J.-D., Coopersmith C.M. (2016). The Third International Consensus Definitions for Sepsis and Septic Shock (Sepsis-3). J. Am. Med. Assoc..

[2] Bauer M., Gerlach H., Vogelmann T., Preissing F., Stiefel J., Adam D. (2020). Mortality in Sepsis and Septic Shock in Europe, North America and Australia between 2009 and 2019—Results from a Systematic Review and Meta-Analysis. Crit. Care.

[3] GBD 2019 Antimicrobial Resistance Collaborators (2022). Global Mortality Associated with 33 Bacterial Pathogens in 2019: A Systematic Analysis for the Global Burden of Disease Study 2019. Lancet.

[4] Kumar A., Roberts D., Wood K.E., Light B., Parrillo J.E., Sharma S., Suppes R., Feinstein D., Zanotti S., Taiberg L. (2006). Duration of Hypotension before Initiation of Effective Antimicrobial Therapy Is the Critical Determinant of Survival in Human Septic Shock. Crit. Care Med..

[5] Im Y., Kang D., Ko R.E., Lee Y.J., Lim S.Y., Park S., Na S.J., Chung C.R., Park M.H., Oh D.K. (2022). Time-to-Antibiotics and Clinical Outcomes in Patients with Sepsis and Septic Shock: A Prospective Nationwide Multicentre Cohort Study. Crit. Care.

[6] Taylor S.P., Anderson W.E., Beam K., Taylor B., Ellerman J., Kowalkowski M.A. (2021). The Association between Antibiotic Delay Intervals and Hospital Mortality among Patients Treated in the Emergency Department for Suspected Sepsis. Crit. Care Med..

[7] Puskarich M.A., Trzeciak S., Shapiro N.I., Arnold R.C., Horton J.M., Studnek J.R., Kline J.A., Jones A.E. (2011). Association between Timing of Antibiotic Administration and Mortality from Septic Shock in Patients Treated with a Quantitative Resuscitation Protocol. Crit. Care Med..

[8] Force I.S.T. (2018). Infectious Diseases Society of America (IDSA) Position Statement: Why IDSA Did Not Endorse the Surviving Sepsis Campaign Guidelines. Clin. Infect. Dis..

[9] Rhee C., Chiotos K., Cosgrove S.E., Heil E.L., Kadri S.S., Kalil A.C., Gilbert D.N., Masur H., Septimus E.J., Sweeney D.A. (2020). Infectious Diseases Society of America Position Paper: Recommended Revisions to the National Severe Sepsis and Septic Shock Early Management Bundle (SEP-1) Sepsis Quality Measure. Clin. Infect. Dis..

[10] Baggs J., Fridkin S.K., Pollack L.A., Srinivasan A., Jernigan J.A. (2016). Estimating National Trends in Inpatient Antibiotic Use among US Hospitals from 2006 to 2012. JAMA Intern. Med..

[11] Al-Dorzi H.M., Eissa A.T., Khan R.M., Harbi S.A.A., Aldabbagh T., Arabi Y.M. (2019). Dosing Errors of Empirical Antibiotics in Critically Ill Patients with Severe Sepsis or Septic Shock: A Prospective Observational Study. Int. J. Health Sci..

[12] De Waele J.J., Lipman J., Akova M., Bassetti M., Dimopoulos G., Kaukonen M., Koulenti D., Martin C., Montravers P., Rello J. (2014). Risk Factors for Target Non-attainment during Empirical Treatment with β-Lactam Antibiotics in Critically Ill Patients. Intensive Care Med..

[13] Casu G.S., Hites M., Jacobs F., Cotton F., Wolff F., Beumier M., de Backer D., Vincent J.-L., Taccone F.S. (2013). Can Changes in Renal Function Predict Variations in Beta-Lactam Concentrations in Septic Patients?. Int. J. Antimicrob. Agents.

[14] Roberts J.A., Joynt G.M., Lee A., Choi G., Bellomo R., Kanji S., Mudaliar M.Y., Peake S.L., Stephens D., Taccone F.S. (2021). The Effect of Renal Replacement Therapy and Antibiotic Dose on Antibiotic Concentrations in Critically Ill Patients: Data from the Multinational Sampling Antibiotics in Renal Replacement Therapy Study. Clin. Infect. Dis..

[15] Choi G., Gomersall C.D., Tian Q., Joynt G.M., Freebairn R., Lipman J. (2009). Principles of Antibacterial Dosing in Continuous Renal Replacement Therapy. Crit. Care Med..

[16] Forrest A., Ballow C.H., Nix D.E., Birmingham M.C., Schentag J.J. (1993). Development of a Population Pharmacokinetic Model and Optimal Sampling Strategies for Intravenous Ciprofloxacin. Antimicrob. Agents Chemother..

[17] Isla A., Rodriguez-Gascon A., Troconiz I.F., Bueno L., Solinis M.A., Maynar J., Sánchez-Izquierdo J.Á., Pedraz J.L. (2008). Population Pharmacokinetics of Meropenem in Critically Ill Patients Undergoing Continuous Renal Replacement Therapy. Clin. Pharmacokinet..

[18] Mahmoud S.H., Shen C. (2017). Augmented Renal Clearance in Critical Illness: An Important Consideration in Drug Dosing. Pharmaceutics.

[19] Udy A.A., Varghese J.M., Altukroni M., Briscoe S., McWhinney B.C., Ungerer J.P., Lipman J., Roberts J.A. (2012). Subtherapeutic Initial Beta-Lactam Concentrations in Select Critically Ill Patients: Association between Augmented Renal Clearance and Low Trough Drug Concentrations. Chest.

[20] Baptista J.P., Sousa E., Martins P.J., Pimentel J.M. (2012). Augmented Renal Clearance in Septic Patients and Implications for Vancomycin Optimisation. Int. J. Antimicrob. Agents.

[21] Herrera-Gutiérrez M.E., Seller-Pérez G., Banderas-Bravo E., Muñoz-Bono J., Lebrón-Gallardo M., Fernandez-Ortega J.F. (2007). Replacement of 24-h Creatinine Clearance by 2-h Creatinine Clearance in Intensive Care Unit Patients: A Single-Centre Study. Intensive Care Med..

[22] Abdul-Aziz M.H., Alffenaar J.C., Bassetti M., Bracht H., Dimopoulos G., Marriott D., Neely M.N., Paiva J.A., Pea F., Sjövall F. (2020). Antimicrobial Therapeutic Drug Monitoring in Critically Ill Adult Patients: A Position Paper. Intensive Care Med..

[23] Gonçalves-Pereira J., Póvoa P. (2011). Antibiotics in Critically Ill Patients: A Systematic Review of the Pharmacokinetics of Beta-Lactams. Crit. Care.

[24] Wong G., Briscoe S., Adnan S., McWhinney B., Ungerer J., Lipman J., Roberts J.A. (2013). Protein Binding of Beta-Lactam Antibiotics in Critically Ill Patients: Can We Successfully Predict Unbound Concentrations?. Antimicrob. Agents Chemother..

[25] Pereira J.G., Fernandes J., Duarte A.R., Fernandes S.M. (2022). β-Lactam Dosing in Critical Patients: A Narrative Review of Optimal Efficacy and the Prevention of Resistance and Toxicity. Antibiotics.

[26] Thompson A., Li F., Gross A.K. (2017). Considerations for Medication Management and Anticoagulation during Continuous Renal Replacement Therapy. AACN Adv. Crit. Care.

[27] Pistolesi V., Morabito S., Di Mario F., Regolisti G., Cantarelli C., Fiaccadori E. (2019). A Guide to Understanding Antimicrobial Drug Dosing in Critically Ill Patients on Renal Replacement Therapy. Antimicrob. Agents Chemother..

[28] Bugge J.F. (2004). Influence of Renal Replacement Therapy on Pharmacokinetics in Critically Ill Patients. Best Pract. Res. Clin. Anaesthesiol..

[29] Scharf C., Liebchen U., Paal M., Taubert M., Vogeser M., Irlbeck M., Zoller M., Schroeder I. (2020). The Higher the Better? Defining the Optimal Beta-Lactam Target for Critically Ill Patients to Reach Infection Resolution and Improve Outcome. J. Intensive Care.

[30] European Medicines Agency (2016). Guideline on the Use of Pharmacokinetics and Pharmacodynamics in the Development of Antimicrobial Medicinal Products.

[31] Blot S.I., Pea F., Lipman J. (2014). The Effect of Pathophysiology on Pharmacokinetics in the Critically Ill Patient—Concepts Appraised by the Example of Antimicrobial Agents. Adv. Drug Deliv. Rev..

[32] Abdulla A., Dijkstra A., Hunfeld N.G.M., Endeman H., Bahmany S., Ewoldt T.M.J., Muller A.E., van Gelder T., Gommers D., Koch B.C.P. (2020). Failure of Target Attainment of Beta-Lactam Antibiotics in Critically Ill Patients and Associated Risk Factors: A Two-Centre Prospective Study (EXPAT). Crit. Care.

[33] Zerbaxa^®^ Ceftolozane and Tazobactam for Injection. https://www.merck.com/product/usa/pi_circulars/z/zerbaxa/zerbaxa_pi.pdf.

[34] Assefa G.M., Roberts J.A., Mohammed S.A., Sime F.B. (2024). What Are the Optimal Pharmacokinetic/Pharmacodynamic Targets for β-Lactamase Inhibitors? A Systematic Review. J. Antimicrob. Chemother..

[35] Guilhaumou R., Benaboud S., Bennis Y., Dahyot-Fizelier C., Dailly E., Gandia P., Goutelle S., Lefeuvre S., Mongardon N., Roger C. (2019). Optimisation of the Treatment with Beta-Lactam Antibiotics in Critically Ill Patients—Guidelines from the French Society of Pharmacology and Therapeutics (Société Française de Pharmacologie et Thérapeutique-SFPT) and the French Society of Anaesthesia and Intensive Care Medicine (Société Française d’Anesthésie et Réanimation-SFAR). Crit. Care.

[36] Zhanel G.G., Chung P., Adam H., Zelenitsky S., Denisuik A., Schweizer F., Lagacé-Wiens P.R.S., Rubinstein E., Gin A.S., Walkty A. (2014). Ceftolozane/Tazobactam: A Novel Cephalosporin/Beta-Lactamase Inhibitor Combination with Activity against Multidrug-Resistant Gram-Negative Bacilli. Drugs.

[37] Merdjan H., Tarral A., Das S., Li J. (2017). Phase 1 Study Assessing the Pharmacokinetic Profile and Safety of Avibactam in Patients with Renal Impairment. J. Clin. Pharmacol..

[38] Wooley M., Miller B., Krishna G., Hershberger E., Chandorkar G. (2014). Impact of Renal Function on the Pharmacokinetics and Safety of Ceftolozane–Tazobactam. Antimicrob. Agents Chemother..

[39] El Nekidy W.S., Al Ali M., Abidi E., Ghazi I.M., Attallah N., El Lababidi R., Mooty M., Ghosn M., Mallat J. (2023). Microbiologic Outcomes of Ceftazidime–Avibactam Dosing in Patients with Sepsis Utilising Renal Replacement Therapies. Hemodial. Int..

[40] Gatti M., Giannella M., Raschi E., Viale P., De Ponti F. (2021). Ceftolozane/Tazobactam Exposure in Critically Ill Patients Undergoing Continuous Renal Replacement Therapy: A PK/PD Approach to Tailor Dosing. J. Antimicrob. Chemother..

[41] Bremmer D.N., Nicolau D.P., Burcham P., Chunduri A., Shidham G., Bauer K.A. (2016). Ceftolozane/Tazobactam Pharmacokinetics in a Critically Ill Adult Receiving Continuous Renal Replacement Therapy. Pharmacotherapy.

[42] El Nekidy W.S., Al Ali M., Abidi E., El Lababidi R., Alrahmany D., Ghazi I.M., Mooty M., Hijazi F., Ghosn M., Mallat J. (2024). Clinical Outcomes of Ceftazidime–Avibactam versus Ceftolozane–Tazobactam in Managing Pseudomonal Infections in Patients Undergoing Renal Replacement Therapy. Antibiotics.

[43] Natesan S., Pai M.P., Lodise T.P. (2017). Determination of Alternative Ceftolozane/Tazobactam Dosing Regimens for Patients with Infections Due to *Pseudomonas aeruginosa* with MIC Values between 4 and 32 mg/L. J. Antimicrob. Chemother..

[44] Butragueño-Laiseca L., Troconiz I.F., Grau S., Campillo N., García X., Padilla B., Fernández S.N., Santiago M.J. (2020). Finding the Dose for Ceftolozane–Tazobactam in Critically Ill Children with and without Acute Kidney Injury. Antibiotics.

[45] Zhanel G.G., Lawson C.D., Adam H., Schweizer F., Zelenitsky S., Lagacé-Wiens P., Denisuik A., Rubinstein E., Gin A.S., Hoban D.J. (2013). Ceftazidime–Avibactam: A Novel Cephalosporin/β-Lactamase Inhibitor Combination. Drugs.

[46] Ulldemolins M., Roberts J.A., Lipman J., Rello J. (2011). Antibiotic Dosing in Multiple Organ Dysfunction Syndrome. Chest.

[47] Food and Drug Administration (2015). Highlights of Prescribing Information. Ceftazidime–Avibactam Package Insert. Allergen. AVYCAZ Safely and Effectively. https://www.accessdata.fda.gov/drugsatfda_docs/label/2015/206494s000lbl.pdf.

[48] Wenzler E., Bunnell K.L., Bleasdale S.C., Benken S., Danziger L.H., Rodvold K.A. (2017). Pharmacokinetics and Dialytic Clearance of Ceftazidime–Avibactam in a Critically Ill Patient on Continuous Venovenous Hemofiltration. Antimicrob. Agents Chemother..

[49] Soukup P., Faust A.C., Edpuganti V., Putnam W.C., McKinnell J.A. (2019). Steady-State Ceftazidime–Avibactam Serum Concentrations and Dosing Recommendations in a Critically Ill Patient Being Treated for *Pseudomonas aeruginosa* Pneumonia and Undergoing Continuous Venovenous Hemodiafiltration. Pharmacother. J. Hum. Pharmacol. Drug Ther..

[50] Shields R.K., Nguyen M.H., Chen L., Press E.G., Kreiswirth B.N., Clancy C.J. (2018). Pneumonia and Renal Replacement Therapy Are Risk Factors for Ceftazidime–Avibactam Treatment Failures and Resistance among Patients with Carbapenem-Resistant *Enterobacteriaceae* Infections. Antimicrob. Agents Chemother..

[51] Shields R.K., Chen L., Cheng S., Chavda K.D., Press E.G., Snyder A., Pandey R., Doi Y., Kreiswirth B.N., Nguyen M.H. (2017). Emergence of Ceftazidime–Avibactam Resistance Due to Plasmid-Borne *blaKPC-3* Mutations during Treatment of Carbapenem-Resistant *Klebsiella pneumoniae* Infections. Antimicrob. Agents Chemother..

[52] Ji Z., Sun K., Li Z., Cheng W., Yang J. (2021). Carbapenem-Resistant *Klebsiella pneumoniae* Osteomyelitis Treated with Ceftazidime–Avibactam in an Infant: A Case Report. Infect. Drug Resist..

[53] Wang W., Wang R., Zhang Y., Zeng L., Kong H., Bai X., Zhang W., Liang T. (2022). Ceftazidime–Avibactam as Salvage Therapy in Paediatric Liver Transplantation Patients with Infections Caused by Carbapenem-Resistant *Enterobacterales*. Infect. Drug Resist..

[54] Perruccio K., Rosaria D., Amico M., Valentina B., Daniela O., Francesca C., Elisabetta C., Paola M., Antonella C., Daniele Z. (2022). Ceftolozane/Tazobactam and Ceftazidime/Avibactam: An Italian Multi-Centre Retrospective Analysis of Safety and Efficacy in Children with Haematologic Malignancies and Multi-Drug Resistant Gram-Negative Bacteria Infections. Pediatr. Infect. Dis. J..

[55] Meng H., Zhao Y., An Q., Zhu B., Cao Z., Lu J. (2023). Use of Ceftazidime–Avibactam for Suspected or Confirmed Carbapenem-Resistant Organisms in Children: A Retrospective Study. Infect. Drug Resist..

[56] Araujo da Silva A.R., Quijada R. (2024). Use of Ceftazidime–Avibactam in Children Admitted to Paediatric Intensive Care Units. Children.

[57] Li D., Yu H., Huang X., Long S., Zhang J. (2023). In Vitro Activity of Ceftazidime–Avibactam, Imipenem–Relebactam, Aztreonam–Avibactam, and Comparators toward Carbapenem-Resistant and Hypervirulent Isolates. Microbiol. Spectr..

[58] Ran X., Chen X., Wang C., Wang H., Xie W., Jing C. (2025). Carbapenem-Resistant *Klebsiella pneumoniae* Infections in Chinese Children: In Vitro Activities of Ceftazidime–Avibactam and Aztreonam–Avibactam against Carbapenemase-Producing Strains in a Two-Centre Study. Front. Cell. Infect. Microbiol..

[59] Ehmann D.E., Jahić H., Ross P.L., Gu R.-F., Hu J., Kern G., Walkup G.K., Fisher S.L. (2012). Avibactam Is a Covalent, Reversible, Non-β-Lactam β-Lactamase Inhibitor. Proc. Natl. Acad. Sci. USA.

[60] Carmeli Y., Cisneros J.M., Paul M., Daikos G.L., Wang M., Torre-Cisneros J., Singer G., Titov I., Gumenchuk I., Zhao Y. (2025). Aztreonam–Avibactam versus Meropenem for the Treatment of Serious Infections Caused by Gram-Negative Bacteria (REVISIT): A Descriptive, Multinational, Open-Label, Phase 3, Randomised Trial. Lancet Infect. Dis..

[61] Li J., Learoyd M., Qiu F., Zhu L., Edeki T. (2016). A Randomised, Phase I Study to Assess the Safety, Tolerability and Pharmacokinetics of Ceftazidime–Avibactam in Healthy Chinese Subjects. Clin. Drug Investig..

[62] Gatti M., Rinaldi M., Gaibani P., Siniscalchi A., Tonetti T., Giannella M., Viale P., Pea F. (2023). A Descriptive Pharmacokinetic/Pharmacodynamic Analysis of Continuous Infusion Ceftazidime–Avibactam for Treating DTR Gram-Negative Infections in a Case Series of Critically Ill Patients Undergoing Continuous Veno-Venous Haemodiafiltration (CVVHDF). J. Crit. Care.

[63] Lodise T.P., O’Donnell J.N., Raja S., Guptill J.T., Zaharoff S., Schwager N., Fowler V.G., Beresnev T., Wall A., Wiegand K. (2022). Safety of Ceftazidime–Avibactam in Combination with Aztreonam (COMBINE) in a Phase I, Open-Label Study in Healthy Adult Volunteers. Antimicrob. Agents Chemother..

[64] Cornely O.A., Cisneros J.M., Torre-Cisneros J., Rodríguez-Hernández M.J., Tallón-Aguilar L., Calbo E., Horcajada J.P., Queckenberg C., Zettelmeyer U., Arenz D. (2020). Pharmacokinetics and Safety of Aztreonam/Avibactam for the Treatment of Complicated Intra-Abdominal Infections in Hospitalised Adults: Results from the REJUVENATE Study. J. Antimicrob. Chemother..

[65] Barbier F., Hraiech S., Kernéis S., Veluppillai N., Pajot O., Poissy J., Roux D., Zahar J.R., French Intensive Care Society (2023). Rationale and Evidence for the Use of New Beta-Lactam/Beta-Lactamase Inhibitor Combinations and Cefiderocol in Critically Ill Patients. Ann. Intensive Care.

[66] Livermore D.M., Mushtaq S., Vickers A., Woodford N. (2023). Activity of Aztreonam/Avibactam Against Metallo-β-Lactamase-Producing *Enterobacterales* from the UK: Impact of Penicillin-Binding Protein-3 Inserts and CMY-42 β-Lactamase in *Escherichia coli*. Int. J. Antimicrob. Agents.

[67] Nichols W.W., Newell P., Critchley I.A., Riccobene T., Das S. (2018). Avibactam Pharmacokinetic/Pharmacodynamic Targets. Antimicrob. Agents Chemother..

[68] Das S., Riccobene T., Carrothers T.J., Wright J.G., MacPherson M., Cristinacce A., McFadyen L., Xie R., Luckey A., Raber S. (2024). Dose Selection for Aztreonam–Avibactam, Including Adjustments for Renal Impairment, for Phase IIa and Phase III Evaluation. Eur. J. Clin. Pharmacol..

[69] Zou C., Wei J., Shan B., Chen X., Wang D., Niu S. (2020). In Vitro Activity of Ceftazidime–Avibactam and Aztreonam–Avibactam Against Carbapenem-Resistant Isolates Collected from Three Secondary Hospitals in Southwest China Between 2018 and 2019. Infect. Drug Resist..

[70] Ilham D., Souad L., Asmae L., Kawtar N., Mohammed T., Nabila S. (2023). Prevalence, Antibiotic Resistance Profile, MBLs Encoding Genes, and Biofilm Formation Among Clinical Carbapenem-Resistant *Enterobacterales* Isolated from Patients in Mohammed VI University Hospital Centre, Morocco. Lett. Appl. Microbiol..

[71] Wu J.Y., Srinivas P., Pogue J.M. (2020). Cefiderocol: A Novel Agent for the Management of Multidrug-Resistant Gram-Negative Organisms. Infect. Dis. Ther..

[72] Nakamura R., Ito-Horiyama T., Takemura M., Toba S., Matsumoto S., Ikehara T., Tsuji M., Sato T., Yamano Y. (2019). In Vivo Pharmacodynamic Study of Cefiderocol, a Novel Parenteral Siderophore Cephalosporin, in Murine Thigh and Lung Infection Models. Antimicrob. Agents Chemother..

[73] Katsube T., Echols R., Arjona Ferreira J.C., Krenz H.K., Berg J.K., Galloway C. (2017). Cefiderocol, a Siderophore Cephalosporin for Gram-Negative Bacterial Infections: Pharmacokinetics and Safety in Subjects with Renal Impairment. J. Clin. Pharmacol..

[74] Katsube T., Echols R., Wajima T. (2019). Pharmacokinetic and Pharmacodynamic Profiles of Cefiderocol, a Novel Siderophore Cephalosporin. Clin. Infect. Dis..

[75] Mornese Pinna S., Corcione S., De Nicolò A., Montrucchio G., Scabini S., Vita D., De Benedetto I., Lupia T., Mula J., Di Perri G. (2022). Pharmacokinetics of Cefiderocol in Critically Ill Patients Receiving Renal Replacement Therapy: A Case Series. Antibiotics.

[76] Shionogi Inc (2021). Fetroja: Cefiderocol for Injection for Intravenous Use, Prescribing Information.

[77] https://www.ema.europa.eu/en/documents/product-information/fetcroja-epar-product-information_en.pdf.

[78] Katsube T., Echols R., Wajima T. (2019). Prediction of Cefiderocol Pharmacokinetics and Probability of Target Attainment in Paediatric Subjects for Proposing Dose Regimens. Open Forum Infect. Dis..

[79] Katsube T., Nicolau D.P., Rodvold K.A., Wunderink R.G., Echols R., Matsunaga Y., Menon A., Portsmouth S., Wajima T. (2021). Intrapulmonary Pharmacokinetic Profile of Cefiderocol in Mechanically Ventilated Patients with Pneumonia. J. Antimicrob. Chemother..

[80] Bottari G., Isabella G., Corrado C., Cairoli S., Marano M., Galaverna F., Stoppa F., Boccieri E., Labbadia R., Cappoli A. (2025). Impact of Continuous Renal Replacement Therapy and CytoSorb on Cefiderocol Pharmacokinetics: “One Size Does Not Fit All”. Int. J. Artif. Organs.

[81] Wunderink R.G., Giamarellos-Bourboulis E.J., Rahav G., Mathers A.J., Bassetti M., Vazquez J., Cornely O.A., Solomkin J., Bhowmick T., Bishara J. (2018). Effect and Safety of Meropenem–Vaborbactam versus Best-Available Therapy in Patients with Carbapenem-Resistant *Enterobacteriaceae* Infections: The TANGO II Randomized Clinical Trial. Infect. Dis. Ther..

[82] Griffith D.C., Sabet M., Tarazi Z., Lomovskaya O., Dudley M.N. (2019). Pharmacokinetics/Pharmacodynamics of Vaborbactam, a Novel β-Lactamase Inhibitor, in Combination with Meropenem. Antimicrob. Agents Chemother..

[83] Griffith D.C., Loutit J.S., Morgan E.E., Keedy K., Persson T., Brown D., Doyle T.B., Steenbergen J.N., Scangarella N.E., Dudley M.N. (2016). Phase 1 Study of the Safety, Tolerability, and Pharmacokinetics of the β-Lactamase Inhibitor Vaborbactam (RPX7009) in Healthy Adult Subjects. Antimicrob. Agents Chemother..

[84] Rubino C.M., Bhavnani S.M., Loutit J.S., Dudley M.N. (2018). Single-Dose Pharmacokinetics and Safety of Meropenem–Vaborbactam in Subjects with Chronic Renal Impairment. Antimicrob. Agents Chemother..

[85] Kufel W.D., Eranki A.P., Paolino K.M., Call A., Miller C.D., Mogle B.T. (2019). In Vivo Pharmacokinetic Analysis of Meropenem/Vaborbactam During Continuous Venovenous Haemodialysis. J. Antimicrob. Chemother..

[86] Sime F.B., Pandey S., Karamujic N., Parker S., Alexander E., Loutit J., Durso S., Griffith D., Lipman J., Wallis S.C. (2018). Ex Vivo Characterisation of Effects of Renal Replacement Therapy Modalities and Settings on Pharmacokinetics of Meropenem and Vaborbactam. Antimicrob. Agents Chemother..

[87] Trang M., Griffith D.C., Bhavnani S.M., Rubino C.M., Ambrose P.G. (2021). Population Pharmacokinetics of Meropenem and Vaborbactam Based on Data from Noninfected Subjects and Infected Patients. Antimicrob. Agents Chemother..

[88] Reed G., Valadez A., Smith B.J., McCreary E.K., Kline E.G., Neely M.N., Squires K.M., Shields R.K., Rhodes N.J. (2025). Population Pharmacokinetics of Meropenem–Vaborbactam in Acutely Ill Hospitalised Patients with Various Degrees of Renal Dysfunction Including Continuous Renal Replacement Therapy. Antimicrob. Agents Chemother..

[89] Fornari C., Arrieta A., Bradley J.S., Tout M., Magalhaes P., Auriol F.K., Borella E., Piana C., Della Pasqua O., Vallespir B.P. (2024). Dose Rationale for the Use of Meropenem/Vaborbactam Combination in Paediatric Patients with Gram-Negative Bacterial Infections. Br. J. Clin. Pharmacol..

[90] Brown M.L., Motsch J., Kaye K.S., File T.M., Boucher H.W., Vendetti N., Aggrey A., Joeng H.K., Tipping R.W., Du J. (2020). Evaluation of Renal Safety Between Imipenem/Relebactam and Colistin Plus Imipenem in Patients with Imipenem-Nonsusceptible Bacterial Infections in the Randomised, Phase III RESTORE-IMI 1 Study. Open Forum Infect. Dis..

[91] Fratoni A.J., Kois A.K., Gluck J.A., Nicolau D.P., Kuti J.L. (2024). Imipenem/Relebactam Pharmacokinetics in Critically Ill Patients Supported on Extracorporeal Membrane Oxygenation. J. Antimicrob. Chemother..

[92] Motsch J., Murta de Oliveira C., Stus V., Köksal I., Lyulko O., Boucher H.W., Kaye K.S., File T.M., Brown M.L., Khan I. (2020). RESTORE-IMI 1: A Multicentre, Randomised, Double-Blind Trial Comparing Efficacy and Safety of Imipenem/Relebactam vs Colistin Plus Imipenem in Patients with Imipenem-Nonsusceptible Bacterial Infections. Clin. Infect. Dis..

[93] Merck & Co., Inc Recarbrio: Package Insert. https://www.merckconnect.com/recarbrio/dosing-administration.

[94] Jang S.M., Infante S., Abdi Pour A. (2020). Drug Dosing Considerations in Critically Ill Patients Receiving Continuous Renal Replacement Therapy. Pharmacy.

[95] Hoff B.M., Maker J.H., Dager W.E., Heintz B.H. (2020). Antibiotic Dosing for Critically Ill Adult Patients Receiving Intermittent Haemodialysis, Prolonged Intermittent Renal Replacement Therapy, and Continuous Renal Replacement Therapy: An Update. Ann. Pharmacother..

[96] Jang S.M., Yessayan L., Dean M., Costello G., Katwaru R., Mueller B.A. (2021). Imipenem/Relebactam Ex Vivo Clearance During Continuous Renal Replacement Therapy. Antibiotics.

[97] DeRyke C.A., Wise M.G., Bauer K.A., Siddiqui F., Young K., Motyl M.R., Sahm D.F. (2025). Antimicrobial Activity of Imipenem/Relebactam and Comparator Agents Against Gram-Negative Isolates Collected from Paediatric Patients: SMART 2018–2022 Global Surveillance. J. Pediatr. Infect. Dis. Soc..

[98] Karlowsky J.A., Lob S.H., Young K., Motyl M.R., Sahm D.F. (2021). In Vitro Activity of Imipenem/Relebactam Against Gram-Negative Bacilli from Paediatric Patients—Study for Monitoring Antimicrobial Resistance Trends (SMART) Global Surveillance Program 2015–2017. J. Pediatr. Infect. Dis. Soc..

[99] White K.C., Serpa-Neto A., Hurford R., Clement P., Laupland K.B., See E., McCullough J., White H., Shekar K., Tabah A. (2023). Sepsis-Associated Acute Kidney Injury in the Intensive Care Unit: Incidence, Patient Characteristics, Timing, Trajectory, Treatment, and Associated Outcomes. A Multicentre, Observational Study. Intensive Care Med..

[100] Papp-Wallace K.M., McLeod S.M., Miller A.A. (2023). Durlobactam, a Broad-Spectrum Serine β-Lactamase Inhibitor, Restores Sulbactam Activity Against *Acinetobacter* Species. Clin. Infect. Dis..

[101] Penwell W.F., Shapiro A.B., Giacobbe R.A., Gu R.-F., Gao N., Thresher J., McLaughlin R.E., Huband M.D., DeJonge B.L.M., Ehmann D.E. (2015). Molecular Mechanisms of Sulbactam Antibacterial Activity and Resistance Determinants in *Acinetobacter baumannii*. Antimicrob. Agents Chemother..

[102] O’Donnell J., Preston R.A., Mamikonyan G., Stone E., Isaacs R. (2019). Pharmacokinetics, Safety, and Tolerability of Intravenous Durlobactam and Sulbactam in Subjects with Renal Impairment and Healthy Matched Control Subjects. Antimicrob. Agents Chemother..

[103] Abouelhassan Y., Shen Y., Chen A., Ye X., Nicolau D.P., Kuti J.L. (2025). Ex Vivo Assessment of Sulbactam–Durlobactam Clearance During Continuous Renal Replacement Therapy to Guide Dosing Recommendations. Antimicrob. Agents Chemother..

[104] Kufel W.D., Zeineddine N., Fouad A., Roenfanz H.F., Shields R.K., Kline E.G., Warner J., Hanrahan K., Kuti J.L. (2025). Pharmacokinetic and Pharmacodynamic Evaluation of Sulbactam–Durlobactam in a Critically Ill Patient on Continuous Venovenous Hemofiltration Infected with Carbapenem-Resistant *Acinetobacter baumannii–calcoaceticus* Complex. Pharmacotherapy.

[105] Kaye K.S., Belley A., Barth P., Matharu T., Krsanac I., Ong C., Kazmierczak K., Stone E., Lademacher C., Sahm D.F. (2022). Effect of Cefepime/Enmetazobactam vs Piperacillin/Tazobactam on Clinical Cure and Microbiological Eradication in Patients with Complicated Urinary Tract Infection or Acute Pyelonephritis: A Randomised Clinical Trial. JAMA.

[106] Keam S.J. (2024). Cefepime/Enmetazobactam: First Approval. Drugs.

[107] Knechtle P., Shapiro S., Morrissey I., De Piano C., Belley A. (2021). Sigmoid Emax Modelling to Define the Fixed Concentration of Enmetazobactam for MIC Testing in Combination with Cefepime. Antimicrob. Agents Chemother..

[108] Fouad A., McGrath C., Buyukyanbolu E., Kuti J.L. (2025). Ex Vivo Assessment and Simulation to Guide Cefepime–Taniborbactam Dosing Recommendations for Patients Receiving Continuous Renal Replacement Therapy. Antimicrob. Agents Chemother..

